# Mepolizumab improves work productivity, activity limitation, symptoms, and rescue medication use in severe eosinophilic asthma

**DOI:** 10.1111/crj.13474

**Published:** 2022-01-26

**Authors:** Frank C. Albers, Daniel J. Bratton, Necdet B. Gunsoy, Sarah M. Cockle, Rafael Alfonso‐Cristancho, Gert‐Jan Braunstahl

**Affiliations:** ^1^ Respiratory Medical Franchise GSK Raleigh North Carolina USA; ^2^ Clinical Statistics GSK Uxbridge UK; ^3^ Value Evidence and Outcomes GSK Uxbridge UK; ^4^ Value Evidence and Outcomes GSK Brentford UK; ^5^ Value Evidence and Outcomes GSK Philadelphia Pennsylvania USA; ^6^ Department of Pulmonary Medicine Franciscus Gasthuis and Vlietland Rotterdam The Netherlands

**Keywords:** activities of daily living, eosinophilic asthma, patient‐reported outcomes, questionnaire, symptom assessment, work productivity

## Abstract

Patients with severe eosinophilic asthma experience daily activity limitations and reduced productivity at work. Using anonymized individual patient‐level data from two previously conducted randomized, double‐blind, placebo‐controlled studies (MENSA [GSK ID:115588/NCT01691521]; MUSCA [GSK ID:200862/NCT02281318]), we investigated the effect of mepolizumab on work productivity, activity limitation, symptoms, and rescue medication use. Patient‐reported outcomes including Work Productivity and Activity Impairment–General Health (WPAI‐GH) scores (impairment percentages, 0%–100%), global activity limitation (scale 1–4), and perceived change in activity limitation (Likert scale 1–7) since the start of the study were analyzed. WPAI‐GH scores from MENSA were analyzed post hoc for employed patients using mixed model repeated measures; global activity limitation and perceived change in activity limitation from MUSCA were analyzed by ordinal logistic regression. Mean changes from baseline in daily asthma symptom scores (scale 0–5) and rescue medication use (occasions/day) were also assessed, via a post hoc meta‐analysis of MENSA and MUSCA. At study end, WPAI‐GH scores indicative of overall work impairment, impairment while working, and activity impairment consistently improved with mepolizumab versus placebo. Overall, 76% versus 54% of patients rated their activity as “much better,” “better,” or “slightly better” since the start of the study with mepolizumab versus placebo. Mepolizumab was associated with numerically larger improvements from baseline in asthma symptoms (treatment difference 0.21–0.29 points) and rescue medication use (treatment difference −0.08 to −0.22 occasions/day) versus placebo. Our results indicate that patients with severe eosinophilic asthma may experience improved activity limitation, work productivity, symptoms, and rescue medication use with mepolizumab.

## 1 INTRODUCTION

Patients with asthma can struggle in their daily activities and ability to work as they cope with numerous symptoms, such as chest tightness, shortness of breath, coughing, wheezing, and difficulty sleeping.^1^ These symptoms can result in frequent and high‐dose rescue medication use, particularly for patients with severe asthma.^1^, ^2^ Those with an eosinophilic phenotype (characterized by persistent airway infiltration with inflammatory eosinophils)^1^ typically have reduced lung function, experience frequent exacerbations, and have poor asthma control despite using high‐dose inhaled corticosteroids plus ≥1 other controller medication (Global Initiative for Asthma Step IV therapy).^2^ They can therefore experience activity limitation, defined as a long‐term reduction in a person's capacity to perform the usual type or amount of age‐appropriate daily activities.^3^ Together with improving symptoms and asthma control, improving work productivity, and minimizing activity limitation are important steps towards addressing the daily disease burden for patients.^2^

Mepolizumab is a targeted and selective anti‐interleukin 5 monoclonal antibody approved as an add‐on treatment for severe eosinophilic asthma in multiple regions worldwide, and for eosinophilic granulomatosis with polyangiitis and hypereosinophilic syndrome in the United States.^4^, ^5^ Clinical trials in patients with severe eosinophilic asthma have demonstrated that compared with placebo, mepolizumab reduces blood eosinophil counts, exacerbation rates, and the need for oral corticosteroids (OCS), in addition to improving lung function and health‐related quality of life.^6^, ^7^, ^8^ MENSA (GSK ID:115588/NCT01691521) and MUSCA (GSK ID:200862/NCT02281318) were Phase III, randomized, double‐blind, placebo‐controlled studies that explored the impact of mepolizumab on exacerbations, quality of life, and other markers of asthma control in patients receiving optimized standard of care plus four‐weekly mepolizumab or placebo for 32 and 24 weeks, respectively.^7^, ^8^ Using anonymized individual patient‐level data from these trials, our analysis describes the effect of the licensed dose of mepolizumab (100‐mg administered subcutaneously [SC]) versus placebo plus optimized standard of care on work productivity, activity limitation, symptoms, and rescue medication use during MENSA and MUSCA.

## 2 REPORT

Work productivity and activity impairment were assessed in MENSA using the Work Productivity and Activity Impairment–General Health (WPAI‐GH) questionnaire^9^ (recall period 7 days). Activity limitation was assessed in MUSCA using two single‐item questionnaires that quantified patients' global rating of activity limitation and global impression of change in activity limitation since the start of the study. In both studies, asthma symptom scores for the previous 24 h and rescue medication (salbutamol/albuterol) use were monitored via daily eDiary tools. Table S1 provides a summary of all outcomes and the methodology used for their analysis. Ethical approval and written informed patient consent were obtained for both parent studies.

## 3 RESULTS AND DISCUSSIONS

Table 1 outlines the baseline demographics and clinical characteristics of MENSA and MUSCA patients included in this analysis. Of the 385 MENSA patients receiving mepolizumab 100‐mg SC or placebo, 186 (48%) were employed and were therefore included in the WPAI‐GH analysis. At baseline, overall work impairment and impairment while working ranged from 26%–33% (Table 1); activity impairment was approximately 38% and the percentage of work time missed was 7%–10%. Direct causes of unemployment or work productivity impairment were not specified. Among MUSCA patients, 37%–39% rated their activity as “limited” or “very limited.” Baseline asthma symptom scores were similar in both studies, with scores ranging from 1.5–1.6 points; patients were using salbutamol/albuterol on 1.5–1.9 occasions/day (Table 1).

**TABLE 1:** Patient baseline demographics and characteristics

	MENSA (*N* = 385)		MUSCA (*N* = 551)	
	Placebo (*n* = 191)	Mepolizumab 100‐mg SC (*n* = 194)	Placebo (*n* = 277)	Mepolizumab 100‐mg SC (*n* = 274)
Age, years	49.2 (14.3)	51.2 (14.6)	52.1 (12.9)	49.8 (14.0)
Females, *n* (%)	107 (56)	116 (60)	176 (64)	149 (54)
BMI, kg/m^2^	28.0 (5.6)	27.6 (6.2)	27.9 (6.2)	28.5 (6.6)
Duration of asthma, years	19.5 (14.6)	20.5 (12.9)	19.6 (15.0)	19.5 (14.6)
Exacerbations in the 12 months before screening	3.6 (2.8)	3.8 (2.7)	2.7 (1.5)	2.9 (1.9)
Asthma symptom score	1.6 (1.2)	1.6 (1.2)	1.5 (1.1)	1.6 (1.1)
Daily salbutamol/albuterol use, occasions/day	1.7 (2.0)	1.9 (2.3)	1.5 (2.0)	1.5 (1.9)
Employed, *n* (%)	99 (52)	87 (45)	n/a	n/a
Full‐time, *n* (%)	67 (68)	59 (68)	n/a	n/a
Part‐time, *n* (%)	27 (27)	26 (30)	n/a	n/a
WPAI‐GH score				
% Work time missed				
*n*	99	86	n/a	n/a
Mean (*SD*) score	7.4 (20.6)	9.7 (24.0)	n/a	n/a
% Impairment while working				
*n*	87	81	n/a	n/a
Mean (*SD*) score	30.9 (27.8)	25.7 (25.0)	n/a	n/a
% Overall work impairment				
*n*	87	80	n/a	n/a
Mean (*SD*) score	33.0 (29.6)	27.9 (27.1)	n/a	n/a
% Activity impairment				
*n*	190	194	n/a	n/a
Mean (*SD*) score	38.5 (26.9)	38.1 (28.7)	n/a	n/a
Global rating of activity limitation, *n* (%)				
Not limited	n/a	n/a	62 (22)	66 (24)
Slightly limited	n/a	n/a	110 (40)	99 (36)
Limited	n/a	n/a	79 (29)	88 (32)
Very limited	n/a	n/a	23 (8)	20 (7)
Missing	n/a	n/a	3 (1)	1 (<1)

*Note*: All values are mean (*SD*) unless otherwise stated; n/a values are owing to data not collected in the parent study.

Abbreviations: BMI, body mass index; n/a, not available; PEF, peak expiratory flow; SC, subcutaneous; WPAI‐GH, Work Productivity and Activity Impairment–General Health.

**FIGURE 1:**
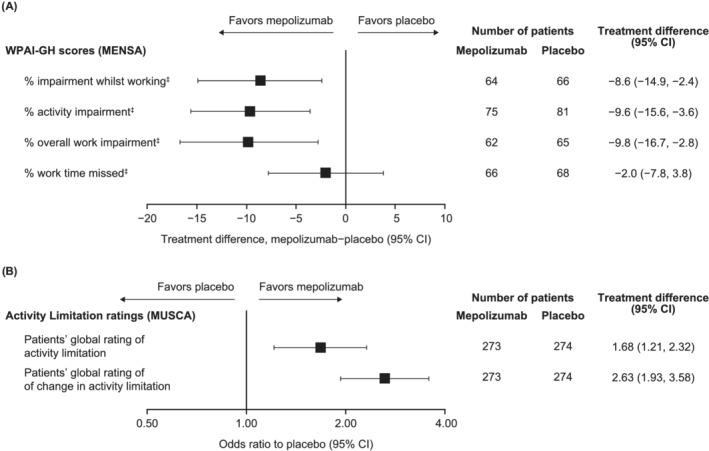
Treatment difference in WPAI‐GH scores (MENSA) and odds ratio for activity limitation ratings (MUSCA) for mepolizumab versus placebo at study end^†^. Four MUSCA patients with missing Global Rating of Activity Limitation data at baseline were not included in this analysis. The analysis of MENSA WPAI‐GH scores was conducted post hoc. ^†^Study end was Week 32 for MENSA and Week 24 for MUSCA; ^‡^due to health. CI, confidence interval; WPAI‐GH, Work Productivity and Activity Impairment–General Health

**FIGURE 2:**
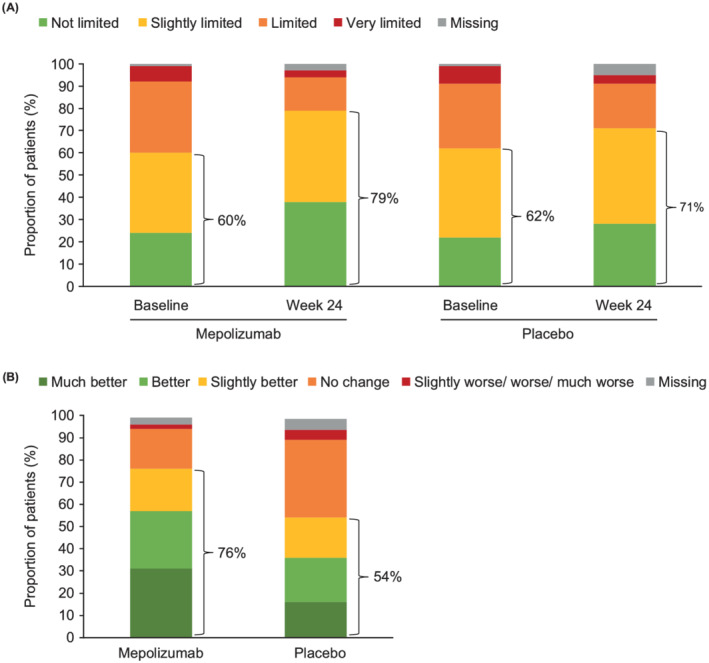
Activity limitation with mepolizumab versus placebo, as measured by (A) Patients' global rating of activity limitation at baseline and Week 24 of the MUSCA study, and (B) global impression of change in activity limitation from the start of the study to Week 24

Following 32 weeks of treatment, we observed consistent improvements with mepolizumab versus placebo in overall work impairment, impairment while working, and activity impairment; a smaller improvement in work time missed was observed (Figure 1A). Patients' perception of their activity limitation also notably improved with mepolizumab versus placebo (Figure 1B). After 24 weeks, 76% versus 54% of patients rated their activity as “much better,” “better,” or “slightly better” since the start of the study with mepolizumab versus placebo (Figure 2). The proportion of patients rating their activity as “not” or “slightly” limited increased an extra 10% from baseline with mepolizumab versus placebo (Figure 2).

**FIGURE 3:**
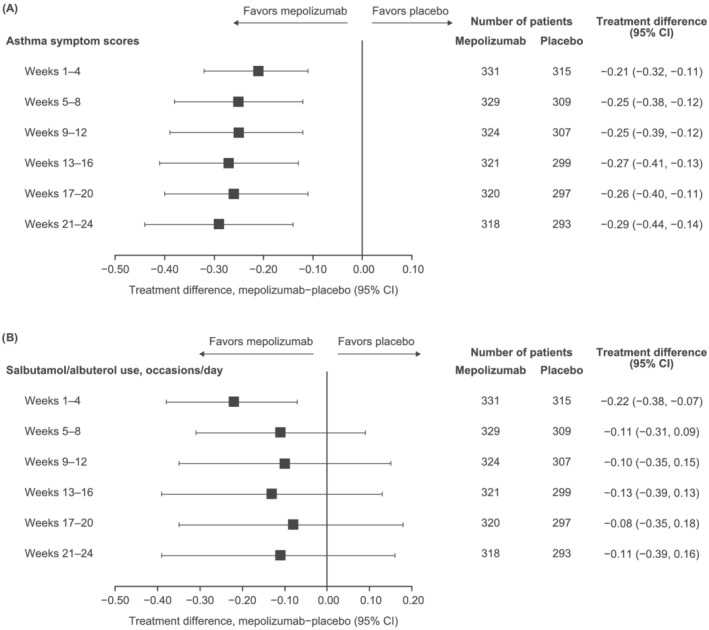
Mean treatment differences in (A) change from baseline in daily asthma symptom scores and (B) daily rescue medication use, by 4‐week period^†^. ^†^Data collected daily using eDiary tool and averaged over 4‐week periods. CI, confidence interval

Across all follow‐up intervals, mean changes from baseline in symptom scores were numerically larger with mepolizumab than placebo (treatment difference 0.21 to 0.29 points; Figure 3). Similarly, mean reductions from baseline in daily salbutamol/albuterol use were numerically larger with mepolizumab versus placebo (treatment difference −0.08 to −0.22 occasions/day; Figure 3).

These results demonstrate that compared with placebo, mepolizumab in addition to optimized standard of care improves work productivity and daily activity limitations for patients with severe eosinophilic asthma. Moreover, mepolizumab appears to improve daily asthma symptoms and the need for rescue medications. Complementary to these findings, a previous analysis of data from the MENSA and MUSCA trials showed that mepolizumab also improves morning peak expiratory flow for patients with severe eosinophilic asthma.^10^

Overall, 33% (*n* = 126/385) and 34% (*n* = 123/364) of the MENSA patients included in this analysis were in full‐time employment (≥35 h/week) at baseline and at study end; this is somewhat lower than the 75% of patients with asthma estimated to be in full‐time employment by a recent multinational survey^11^ and is likely reflective of the increased symptom severity associated with severe eosinophilic asthma. Although only a small difference in work time missed was observed from baseline to follow‐up, enabling patients to increase their productivity at work may lead to indirect cost benefits for employers and for society.^12^ It is worth noting that owing to the post hoc and exploratory nature of these analyses, statistical significance tests were not performed. Moreover, although the WPAI‐GH questionnaire is widely used for assessing work productivity and activity impairment across a range of chronic conditions, it has not been validated specifically for use in patients with asthma. Finally, although both trials demonstrated improvements, real‐world analyses could identify any longer‐term impact of mepolizumab on daily disease burden.

## 4 CONCLUSIONS

Patients with severe eosinophilic asthma may benefit from improved symptoms with mepolizumab, which may in turn lead to improvements in work productivity, activity limitation, and rescue medication use. These data complement the results of the MENSA and MUSCA studies, which showed reductions in exacerbations and hospitalizations with mepolizumab. Moreover, they demonstrate reductions in daily disease burden with optimized standard of care plus mepolizumab and may be an important consideration for healthcare practitioners in their clinical decision making.

## FUNDING INFORMATION

All analyses and the parent studies (MENSA, GSK ID: 115588/NCT01691521; MUSCA, GSK ID: 200862/NCT02281318) were funded by GlaxoSmithKline (GSK).

## CONFLICT OF INTEREST

FCA is an employee of Avillion US, Inc., and a former employee of GSK with stock/stock options in GSK. DJB, SMC, and RAC are all employees of GSK and hold stock/stock options in GSK. NBG is an employee of AbbVie Ltd and a former employee of GSK. GJB has received research grants from GSK, Novartis, Chiesi, and AstraZeneca; personal fees from ALK‐Abello, Sanofi, Novartis, Chiesi, and AstraZeneca.

## AUTHOR CONTRIBUTIONS

FCA, DJB, NBG, SMC, and RAC all contributed to the conception and design of the analysis. GJB was involved in the acquisition of data. All authors were involved in the analysis and interpretation of the data, critically revised the manuscript for intellectual content, gave final approval of the version to be published, and agreed to be accountable for all aspects of the work.

## ETHICS STATEMENT

Ethical approval and written informed patient consent were obtained for the parent studies on which this analysis is based.

## Supporting information


**Table S1.** Description of patient reported outcomes tools

## Data Availability

The data that support the findings of this study are available on request from the corresponding author. The data are not publicly available due to privacy or ethical restrictions.

## References

[1] Chung KF , Wenzel SE , Brozek JL , et al. International ERS/ATS guidelines on definition, evaluation and treatment of severe asthma. Eur Respir J. 2014;43(2):343‐373. 24337046 10.1183/09031936.00202013

[2] Global Initiative for Asthma . Global strategy for asthma management and prevention 2019. 2019. https://ginasthma.org/wp-content/uploads/2019/06/GINA-2019-main-report-June-2019-wms.pdf. Accessed March 1, 2020.

[3] Bogaert P , Van Oyen H , Beluche I , Cambois E , Robine J‐M . The use of the global activity limitation Indicator and healthy life years by member states and the European Commission. Arch Public Health. 2018;76(1):30. 29988309 10.1186/s13690-018-0279-z PMC6022353

[4] European Medicines Agency . Mepolizumab (NUCALA) summary of product characteristics. 2019. https://www.ema.europa.eu/en/documents/product-information/nucala-epar-product-information_en.pdf. Accessed March 1, 2020.

[5] Food and Drug Administration . Mepolizumab (NUCALA) highlights of prescribing information. 2020. https://gskpro.com/content/dam/global/hcpportal/en_US/Prescribing_Information/Nucala/pdf/NUCALA‐PI‐PIL‐IFU‐COMBINED.PDF. Accessed March 15, 2021.

[6] Emma R , Morjaria JB , Fuochi V , Polosa R , Caruso M . Mepolizumab in the management of severe eosinophilic asthma in adults: current evidence and practical experience. Ther Adv Respir Dis. 2018;12:1‐12, 175346661880849. 10.1177/1753466618808490 PMC6204623 30354852

[7] Ortega HG , Liu MC , Pavord ID , et al. Mepolizumab treatment in patients with severe eosinophilic asthma. N Engl J Med. 2014;371(13):1198‐1207. 25199059 10.1056/NEJMoa1403290

[8] Chupp GL , Bradford ES , Albers FC , et al. Efficacy of mepolizumab add‐on therapy on health‐related quality of life and markers of asthma control in severe eosinophilic asthma (MUSCA): a randomised, double‐blind, placebo‐controlled, parallel‐group, multicentre, phase 3b trial. Lancet Respir Med. 2017;5(5):390‐400. 28395936 10.1016/S2213-2600(17)30125-X

[9] Reilly MC , Zbrozek AS , Dukes EM . The validity and reproducibility of a work productivity and activity impairment instrument. PharmacoEconomics. 1993;4(5):353‐365. 10146874 10.2165/00019053-199304050-00006

[10] Ortega H , Menzies‐Gow A , Llanos J‐P , et al. Rapid and consistent improvements in morning PEF in patients with severe eosinophilic asthma treated with mepolizumab. Adv Ther. 2018;35(7):1059‐1068. 29949045 10.1007/s12325-018-0727-8 PMC11343775

[11] Gruffydd‐Jones K , Thomas M , Roman‐Rodríguez M , et al. Asthma impacts on workplace productivity in employed patients who are symptomatic despite background therapy: a multinational survey. J Asthma Allergy. 2019;12:183‐194. 31372003 10.2147/JAA.S204278 PMC6636188

[12] Nunes C , Pereira AM , Morais‐Almeida M . Asthma costs and social impact. Asthma Res Pract. 2017;3(1):1. 28078100 10.1186/s40733-016-0029-3 PMC5219738

