# Supplementary material for: Mepolizumab improves work productivity, activity limitation, symptoms, and rescue medication use in severe eosinophilic asthma
Source: Clin Respir J. 2022 Jan 26;16(3):252–8. doi: 10.1111/crj.13474 (PMC9060075; doi:10.1111/crj.13474)
Supplement: Supplementary file 1 — Table S1. Description of patient reported outcomes tools [file CRJ-16-252-s001.docx]

**Supplementary materials**

**Mepolizumab improves work productivity, activity limitation, symptoms, and rescue medication use in severe eosinophilic asthma**

Albers *et al.*

**Corresponding author:** Rafael Alfonso-Cristancho, MD, PhD, MSc

Address: Global Medical - Value Evidence and Outcomes, GSK, Collegeville, PA, USA

Tel: +1-(610)-917-7000

Email: rafael.x.alfonso@gsk.com

**Contents**

Table S1: Description of patient-reported outcomes

**Table S1:** Description of patient reported outcomes tools

| **Questionnaire/ Study** | **Description** | **Description of response options** | **Description of analyses** |
| --- | --- | --- | --- |
| WPAI-GH/ MENSA | Comprised of 6 questions which addressed absenteeism, presenteeism (reduced effectiveness while working), overall work productivity loss (absenteeism plus presenteeism), and activity impairment,  completed at baseline and at each  4-weekly visit | WPAI:GH outcomes were scored as impairment percentages, with a higher percentage indicating greater impairment and less productivity.  Activity impairment was rated on a scale of 0–10, with 0 being ‘health problems had no effect on my work/doing my daily activities’, and 10 being ‘health problems completely prevented me from working/doing my daily activities’ | Exploratory, post hoc analysis  (not pre-defined):  Scores at study end were analyzed for employed patients using mixed model repeated measures, adjusted for baseline score, geographical region, gender, age, treatment group, and visit; interaction terms were visit by baseline score and visit by treatment group. Baseline maintenance OCS therapy, exacerbations in the prior year (2, 3, 4+), and baseline pre-bronchodilator % predicted FEV_1_ were included as covariates |
| Global rating of activity limitation/ MUSCA | Patients rated their activity limitation since the start of the study using the scale provided, at Weeks 4, 12, 20, and 24 | Activity limitation was rated on a 4-point scale  (1–4): ‘not limited’; ‘slightly limited’; ‘limited’; ‘very limited’ | Pre-specified analysis:  Scores at study end were analyzed by ordinal logistic regression adjusting for treatment group, region, baseline maintenance OCS therapy, exacerbations in the prior year (2, 3, 4+) and baseline % predicted FEV_1_. For analysis purposes, ‘very limited’ and ‘missing’ were combined into one category |
| Global impression of change in activity limitation/ MUSCA | Patients rated their impression of change in activity since the start of the study using the scale provided, at Weeks 4, 12, 20, and 24 | Global impression of change was rated on a  7-point Likert scale (1–7): ‘much better’; ‘better’; ‘slightly better’; ’worse’; ‘slightly worse’; ‘much worse’ | Pre-specified analysis: Scores at study end were analyzed by ordinal logistic regression adjusting for treatment group, region, global rating of activity limitation at baseline, baseline maintenance OCS therapy, exacerbations in the prior year (2, 3, 4+) and baseline % predicted FEV_1_. For analysis purposes, ‘much worse’ and ‘missing’ were combined into one category |
| Asthma Symptoms/ MENSA and MUSCA | Patients recorded an asthma symptom score for the past 24 hours, using the scale provided | Symptoms were rated on a 6-point scale (0–5):  ‘no symptoms during the previous 24-hours’; ‘symptoms for one short period during the previous 24-hours’; ‘Symptoms for two or more short periods during the previous 24-hours’; ‘symptoms for most of the previous 24-hours which did not affect my normal daily activities’; ‘symptoms for most of the previous 24-hours, which did affect my normal daily activities’; ‘symptoms so severe that I could not go to work/school or perform normal daily activities’ | Post hoc meta-analysis:  Daily scores were averaged over 4-week periods. Analysis of change from baseline in mean daily asthma symptom scores was performed using mixed model repeated measures with covariates of study ID, baseline, baseline maintenance OCS therapy (OCS/ no OCS), exacerbations in the year prior to the study (as an ordinal variable; 2, 3, ≥4), baseline % predicted pre-bronchodilator FEV_1_, treatment group, and visit. Interaction terms for visit by baseline and visit by treatment group were applied |
| Rescue medication use/ MENSA and MUSCA | Salbutamol/albuterol use was recorded daily | Least squares mean change from baseline in daily salbutamol/albuterol use (occasions/day) | Post hoc meta-analysis:  Daily rescue medication use averaged over 4-week periods. Analysis of change from baseline in mean daily salbutamol/albuterol use was performed using mixed model repeated measures with covariates of study ID, baseline, baseline maintenance OCS therapy (OCS/ no OCS), exacerbations in the year prior to the study (as an ordinal variable; 2, 3, ≥4), baseline % predicted pre-bronchodilator FEV_1_, treatment group, and visit. Interaction terms for visit by baseline and visit by treatment group were applied |

FEV_1_, forced expiratory volume in 1 second; WPAI-GH, Work Productivity and Activity Impairment – General Health
